# The lack of paid sick leave as a barrier to cancer screening and medical care-seeking: results from the National Health Interview Survey

**DOI:** 10.1186/1471-2458-12-520

**Published:** 2012-07-12

**Authors:** Lucy A Peipins, Ashwini Soman, Zahava Berkowitz, Mary C White

**Affiliations:** 1 Epidemiology and Applied Research Branch, DCPC, CDC, 4770 Buford Highway, NE, Mailstop K-55, Atlanta, GA, 30341-3717, USA; 2 Northrop Grumman, 3375 Northeast Expressway, Atlanta, GA, 30341, USA

**Keywords:** Cancer screening, Pap test, Mammography, FOBT, Colonoscopy, Paid sick leave, Health benefits

## Abstract

**Background:**

Preventive health care services, such as cancer screening can be particularly vulnerable to a lack of paid leave from work since care is not being sought for illness or symptoms. We first describe the prevalence of paid sick leave by broad occupational categories and then examine the association between access to paid sick leave and cancer testing and medical care-seeking in the U.S. workforce.

**Methods:**

Data from the 2008 National Health Interview survey were analyzed by using paid sick leave status and other health-related factors to describe the proportion of U.S. workers undergoing mammography, Pap testing, endoscopy, fecal occult blood test (FOBT), and medical-care seeking.

**Results:**

More than 48 million individuals (38%) in an estimated U.S. working population of 127 million did not have paid sick leave in 2008. The percentage of workers who underwent mammography, Pap test, endoscopy at recommended intervals, had seen a doctor during the previous 12 months or had at least one visit to a health care provider during the previous 12 months was significantly higher among those with paid sick leave compared with those without sick leave after controlling for sociodemographic and health-care-related factors.

**Conclusions:**

Lack of paid sick leave appears to be a potential barrier to obtaining preventive medical care and is a societal benefit that is potentially amenable to change.

## Background

Paid sick leave is paid time taken off from work by individuals to attend to their own or their family member’s illness or other medical needs without loss of pay or job loss. Paid sick leave in the Unites States is a provision by the employer and not an insurance option. Currently in the United States there are no federal legal requirements for paid sick leave [1]. The Federal Family and Medical Leave Act (FMLA) provides up to 12 weeks of unpaid leave for specified medical conditions for employees of companies with 50 or more employees [2], but FMLA does not apply to workers who need time off for routine or preventive medical care. Both San Francisco and Washington, DC have passed legislation guaranteeing paid sick leave to workers in their cities. In addition, measures providing sick leave have passed in Milwaukee, WI and Seattle, WA but have not yet been enacted [3].

Concern about the lack of paid sick leave was heightened during the 2009 H1N1 influenza outbreak when the Centers for Disease Control and Prevention recommended that workers remain at home if they were sick with flu-like symptoms to control the spread of infection [4], and emergency legislation guaranteeing temporary sick leave was introduced in the House of Representatives [5]. In addition to the potential for reducing the spread of infection, the ability to take sick leave is likely to have an effect on a much wider range of health conditions and care-seeking both for workers and their families.

Preventive health services, including cancer screening, can be particularly vulnerable to a lack of paid leave since, by definition, preventive care is not sought for illness or symptoms. The United States Preventive Services Task Force (USPSTF) and the American Cancer Society recommend regular screening for the prevention of breast, colorectal and cervical cancers for early detection or removal of precancerous lesions [6,7]. However, screening rates for breast, cervical, and colorectal cancer in the US remain lower for people with lower income and education, without health insurance, and Hispanic ethnicity [7-10]. The USPSTF also recommends screening for high blood pressure and further screening for diabetes for those with high blood pressure [11]. This screening is typically part of a medical care visit.

Although the lack of health insurance coverage and access to preventive care have been broadly examined, [12-15] we are not aware of research to assess the effect of paid sick leave on the use of cancer screening services. In 2008, the Task Force on Community Preventive Services completed a systematic review of research on client-directed interventions to increase cancer screening [16]. The research examined did not address paid sick leave but did include other efforts to reduce out-of-pocket expenses. The Task Force concluded that there was sufficient evidence to show that reducing out-of-pocket costs increased the use of mammography but the evidence was judged insufficient to determine the effectiveness of similar interventions for cervical or colorectal cancer screenings. The aims of this analysis are to (1) describe the prevalence of paid sick leave by broad occupational categories and other occupationally-related groupings and (2) examine the association between access to paid sick leave and cancer testing and medical care-seeking in the U.S. working population.

## Methods

### Study population

We used data from the 2008 National Health Interview Survey (NHIS), a multi-purpose health survey of a probability-based sample of the U.S. civilian noninstitutionalized population conducted by the CDC’s National Center for Health Statistics (NCHS). The majority of the interviews were conducted in person by trained interviewers from the U.S. Census Bureau, and 25% were completed by telephone. The interviewed sample for 2008 consisted of 74,236 persons in 29,421 families from 28,790 households yielding a household response rate of approximately 85%, a conditional sample adult response rate of 74%, and a final adult sample size of 11,826 with a sample adult response rate of 63% [17].

**Figure 1:**
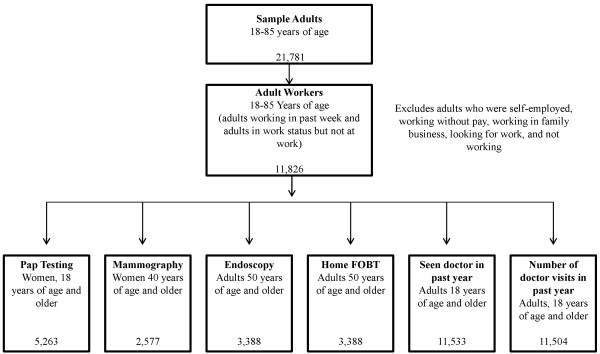
Study samples flow chart, NHIS 2008.

The focus of this analysis was currently employed adults who were 18 years of age and older. This group included adults currently working for pay at a job or business in the prior week or adults working at a job or business but not at work in the prior week. We excluded workers who were self-employed, working without pay, working in a family business, looking for work, or not working (Figure 1).

### Occupational characteristics

Respondents were asked about the kind of work they did (occupation) and the current job or work situation (employed by a private company or federal, state or local government). Two-digit codes based on the Standard Occupational Classification [18] were assigned to each verbatim response by NCHS [19]. We collapsed the occupations into 5 general categories that included management occupations (codes 01–04), professional/technical occupations (codes 05–31), service occupations (codes 32–52), sales and office administrative support occupations (codes 53–64), and a general production category that included construction, production, transportation and maintenance occupations as well as farming, forestry and fishing occupations (codes 65–93).

Information was obtained on the number of people who worked at the respondent’s current job location. The possible response categories of 1–9 employees, 10–24 employees, 25–49 employees, 50–99 employees, 100–249 employees, 250–499 employees, 500–999 employees and 1,000 or more employees were collapsed into 4 groups (Table 1). Currently working respondents reported how many years they had worked at a main job or business. Years at work were categorized as 0–1, 2–5, 6–15 and 16 or more. Respondents answered ‘yes’ or ‘no’ to the question, “Do you have paid sick leave on this main job or business?”

**Table 1:** Percent of U.S. workforce with paid sick leave by occupational characteristics, NHIS, 2008

**Characteristics**	**NHIS sample**	**Estimated # of U.S. workers**	% with paid sick leave*	**95% CI**	**Estimated # of workers with no sick leave**
All workers	11,826	127,067,000	61.9	60.7-63.1	48,352,000
**Occupation**					
Management	874	10,120,800	81.1	77.5-84.2	1,915,200
Professional/Technical	3,281	35,349,700	79.0	77.1-80.7	7,442,100
Services	1,997	21,028,300	41.2	38.3-44.1	12,373,000
Sales/Office	2,873	30,406,100	64.3	62.3-66.3	10,843,800
Production**	2,423	27,492,200	53.9	51.4-56.4	14,818,500
**Class of worker**					
Private	9,577	104,319,000	57.0	55.7-58.3	44,858,100
Federal	306	3,063,100	91.5	86.6-94.7	260,200
State	858	8,967,500	81.5	77.5-85.0	1,655,200
Local	975	10,717,600	85.3	82.2-87.9	1,578,500
**Years on the job**					
0-1	3,250	35,737,500	45.1	42.9-47.3	19,632,200
2-5	3,542	38,651,600	60.3	58.1-62.4	15,353,000
6-15	3,115	32,914,500	71.9	69.8-73.9	9,236,480
16+	1,704	18,662,600	79.4	76.8-81.7	3,854,130
**Number of employees**					
< 10	2,073	22,388,100	41.6	39.0-44.1	13,086,000
10-49	3,084	34,408,900	53.1	50.8-55.4	16,136,000
50-249	2,878	31,806,300	69.4	67.0-71.7	9,737,850
250+	2,485	32,820,100	79.4	77.4-81.3	6,760,420

* Percentages are weighted to the population of workers.

** Production category includes Production, Agricultural, Forest and Fishery workers.

CI indicates confidence interval.

Totals in categories may not sum to all worker totals because of missing and unknown values.

### Cancer tests and medical care seeking

Respondents were asked if they had ever had a colorectal exam, the type of exam, when they had the exam and the reasons for the exam. We classified respondents who reported having had a colonoscopy during the past 10 years or sigmoidoscopy during the past 5 years for any reason as having had an endoscopy within recommended screening guidelines. Although FOBT is currently recommended with sigmoidoscopy [6], the use of sigmoidoscopy represents only a small fraction of endoscopic screening procedures, and this recommendation in 2008 may not be reflected in the data used for this analysis. We used the definition of screening by sigmoidoscopy during the past 5 years to permit comparisons with other published estimates. In addition, respondents were asked if they had ever used an FOBT home kit, and the date of their most recent test. Respondents, who had never had this test or had not had one during the prior year as recommended by national guidelines, were classified as not having the test. Women were asked if they had had a mammography and a Pap smear or Pap test, when they had the tests and the reasons for the test. Women who reported having had a mammogram during the prior 2 years or a Pap test during the prior 3 years as part of a routine exam were classified as having had a mammogram or Pap test respectively [6].

Respondents were asked if they had seen or talked to a general practice, internal medicine or family doctor during the prior 12 months and how many times during the prior 12 months they had seen a doctor or other health care professional in a doctor’s office, clinic or location other than a hospital, emergency room, or dental office or spoken to one by telephone. For this analysis we dichotomized the number of doctor visits as no visits versus one or more visits during the prior 12 months.

### Age groups and gender

For analyses of cancer testing, we included working women 40 years and older in the analysis of mammography. During the time of this survey, recommendations for mammography included women from age 40 to 49 years [20]. All adult working women (18 years of age or older) were included in analyses of Pap testing. Colorectal cancer analyses (endoscopy and FOBT) focused on adults 50 years of age or older. Analyses of the outcomes of those individuals who had seen or spoken with a doctor and the number of visits included all working adults 18 years of age and older. Figure 1 presents a chart of population sub-groups for analyses. We assumed that most adults who were healthy enough to work could potentially benefit from early cancer detection, regardless of age, and therefore we did not apply an upper age limit for the use of any cancer screening test.

### Other covariates

All variables were self-reported. These included age (classified by 10 year age groups), education (less than high school, high school or GED, some college and college graduates), race/ethnicity (Hispanic, non-Hispanic white, non-Hispanic black, and non-Hispanic other), poverty ratio (<100%, 100% to <200%, 200% to <400%, 400% or more), insurance status (private, public only, private and public, not covered and unknown), usual source of medical care (yes, no, and only emergency room care) and marital status (never married, married/partnered, and widowed/divorced). Missing data for income was imputed by using multiple imputation [17].

### Statistical analysis

We used descriptive statistics to examine the distribution of occupational characteristics of the U.S. workforce with and without paid sick leave. In addition, we used the chi-square test to examine the association of having paid sick leave with the uptake of various cancer tests, the number of physician visits and whether members of this population have been seen by a doctor during the prior year. We used six multivariate logistic regression models that show the association between sick leave status and various socio-demographic characteristics with each of the cancer tests, number of physician visits, and whether members saw a doctor during the prior year. To enable easy interpretation of the models’ results, we computed and presented adjusted percentages (predicted margins), which are derived from the logistic regression model [21]. Overall associations were assessed with the Wald F statistic, and differences between categories within each adjusted variable were tested using general linear contrasts of the percentages.

To generalize the results to the population, each respondent was assigned a sampling weight. The weights accounted for selection probability and non-response. A P*-*value of less than 0.05 was considered statistically significant. We considered an estimate to be unstable and recommend caution in interpretation if the relative standard error, (calculated as [standard error/estimated percentage] x 100), was more than 30%. All statistical analyses were performed by using SAS 9.2 with SUDAAN release 10 (Research Triangle Institute, Research Triangle Park, NC) to adjust for the complex sampling design of the NHIS.

## Results

More than 48 million currently employed U.S. workers reported having no paid sick leave at their main job or business (Table 1). The percentage of workers with paid sick leave varied by class of worker, years on the job and number of employees at the respondent’s location of work. Service occupations had the lowest percentage of workers with paid sick leave (41%), and management workers had the highest percentage (81%) among occupational categories. The percentage of workers with paid sick leave was lower among private vs. all levels of the public sector. As years on the job and number of employees in a work location increased, the percentage of workers with paid sick leave increased.

Table 2 presents the relationship between having paid sick leave and cancer testing and medical care seeking. The percentage of workers who underwent mammography, Pap test, endoscopy at recommended intervals, had seen a doctor during the prior 12 months or had at least one visit to a health care provider during the prior 12 months was significantly higher among those with paid sick leave as compared with those without sick leave. The percentage of workers who reported having an FOBT within the prior year was less than 10% and did not vary by sick leave status.

**Table 2:** Percentages and 95% CIs of U.S. workers undergoing cancer tests and medical care-seeking by paid sick leave, NHIS, 2008

		**Paid Sick Leave**				
**Cancer test**	**n**	**Has sick leave**		**Doesn't have sick leave**		p*
		**%**	**95% CI**	**%**	**95% CI**	
Mammography	2,555	83.6	81.5-85.5	75.8	72.1-79.2	<0.001
Pap Test	5,218	89.9	88.7-91.0	86.4	84.5-88.1	<0.001
Endoscopy	3,224	52.7	50.1-55.3	43.1	39.7-46.5	<0.001
Home FOBT	3,208	9.2	7.8-10.8	9.7	7.9-11.9	0.68
# physician visits	11,504	84.0	82.8-85.2	72.0	70.3-73.7	<0.001
in past year						
Seen doctor in	11,533	69.1	67.6-70.4	57.9	56.1-59.8	<0.001
past year						

FOBT, fecal occult blood test; CI, confidence interval;

Mammography within past 2 years, women age >39.

Endoscopy for adult workers within past 5 years for flexible sigmoidoscopy or 10 years for colonoscopy.

Home FOBT for adult workers within past year age > 49.

# physician visits and seen doctor in past 12 months for all workers.

p* values are based on an chi square test for association.

After adjusting for sociodemographic and health related characteristics (Table 3), the associations between paid sick leave and mammography, Pap test and endoscopy remained statistically significant. The unadjusted and adjusted proportions of cancer tests by sick leave were quite similar. Working women 40 years of age and older with sick leave were more likely to have had a mammogram within the prior 2 years (83.3%; 95% CI, 81.2–85.2) than those without sick leave (77.0%; 95% CI, 72.9–79.9). No associations found between age, education, poverty ratio, health insurance status, race/ethnicity and mammography use. However, associations still remained for marital status and having a usual source for medical care. Married or partnered workers were more likely to have had a mammography than those who were widowed or divorced (83.2% vs 77.1%, p < 0.01) adjusting for covariates. Workers without a usual source of care were less likely to report a mammogram (57.1%; 95% CI, 47.5–66.2) than workers who had a usual source of care (82.7%; 95% CI, 81.0–84.3).

**Table 3:** Adjusted population percentages and 95% CIs of U.S. workers undergoing cancer tests, NHIS, 2008

**Characteristics**	**Mammography (n = 2,545)**			**Pap Test (n = 4,505)**			**Endoscopy (n = 3,210)**			**Home FOBT (n = 3,194)**		
	PM*	**95% CI**	P**	PM*	**95% CI**	P**	PM*	**95% CI**	P**	PM*	**95% CI**	P**
**Sick leave**												
Yes	83.3	81.2-85.2	<0.001	91.9	90.6-93.0	<0.04	52.5	49.9-55.0	<0.001	9.3	8.0-10.9	0.83
No	77.0	72.9-79.9		89.9	88.1-91.4		43.5	40.1-47.1		9.6	7.7-11.8	
**Age years**												
18-29	-		0.29	97.0	95.5-98.0	<0.001	-		<0.001	-		<0.001
30-39	-			91.6	89.7-93.1		-			-		
40-49	79.6	76.6-82.3		88.4	86.2-90.3		-			-		
50-59	81.5	78.7-83.1		87.3	84.3-89.8		46.0	43.5-48.6		7.8	6.6-9.2	
60-69	84.4	81.2-88.0		83.1	78.2-87.0		56.9	53.0-60.6		12.7	10.4-15.3	
70+	84.0	74.2-90.4		70.1	55.5-81.5		60.7	52.7-68.2		13.7	9.5-19.4	
**Race/ethnicity**												
Hispanic	80.7	77.0-84.0	0.48	90.4	88.0-92.3	0.90	49.2	44.9-53.6	0.79	9.4	7.3-12.2	0.69
Non-Hispanic White	80.8	78.3-83.1		92.2	90.9-93.4		50.5	47.6-53.4		9.6	8.1-11.3	
Non-Hispanic Black	81.2	76.1-85.4		88.0	84.6-90.7		46.5	40.9-52.2		7.9	5.5-11.3	
Non-Hispanic Asian	87.3	80.3-92.1		90.9	86.7-93.8		51.3	42.9-59.6		10.4	6.0-17.4	
Non-Hispanic Other	81.9	64.6-91.9		94.1	85.5-97.7		48.2	31.9-64.8		14.8	6.6-30.0	
**Marital status**												
Never married	77.7	71.6-82.8	0.00	91.5	89.1-93.5	0.01	42.5	36.0-49.2	0.002	9.1	6.1-13.3	0.02
Married/partnered	83.2	80.9-85.3		92.0	90.6-93.1		51.7	49.0-54.3		8.5	7.2-10.1	
Widowed/Divorced	77.1	74.0-80.0		88.2	85.8-90.2		44.8	41.4-48.2		12.2	10.2-14.7	
**Education**												
< high school	80.5	77.5-83.2	0.08	91.8	90.0-93.3	0.70	49.8	46.1-53.5	0.51	10.2	8.3-12.6	0.80
High school/GED	79.8	75.7-83.4		90.9	88.7-92.7		50.3	46.1-54.5		8.9	6.7-11.8	
Some college	84.3	80.5-87.6		91.4	89.1-93.2		49.4	44.9-53.9		8.6	6.5-11.3	
College graduate	77.8	72.2-82.5		89.7	87.1-91.9		46.0	41.0-51.2		8.9	6.5-12.0	
missing	86.5	80.5-91.0		91.4	88.0-94.0		53.6	46.9-60.2		10.4	7.2-14.9	
**Poverty ratio**												
< 100%	84.0	78.3-88.4	0.20	92.1	88.8-94.5	0.71	43.3	37.3-49.4	0.12	6.7	4.4-10.1	0.14
100% to <200%	76.7	71.6-81.1		89.9	87.3-92.0		52.1	46.5-57.6		11.5	8.5-15.3	
200% to <400%	82.2	78.9-85.1		91.3	89.3-93.0		52.0	47.7-56.3		10.4	8.1-13.4	
400% or more	82.4	79.1-85.3		90.8	89.0-92.3		49.8	46.1-53.4		9.7	7.6-12.2	
Unknown	80.5	76.5-84.1		91.7	89.5-93.5		48.2	43.5-52.9		7.9	5.9-10.6	
**Health insurance**												
Private	80.4	76.9-83.5	0.25	90.4	88.3-92.1	0.2	48.2	44.6-51.8	0.44	9.9	7.9-12.2	0.76
Public only	79.4	75.1-83.2		91.8	89.6-93.6		51.6	47.1-55.9		8.9	7.0-11.2	
Public and Private	84.4	81.4-87.0		90.7	88.6-92.4		51.1	47.1-55.1		10.1	7.8-12.9	
Not covered	80.0	75.6-83.9		91.9	89.6-93.7		46.7	41.8-51.6		8.2	6.0-11.1	
Unknown	78.1	50.6-92.5		97.3	91.9-99.1		51.0	32.5-69.2		6.9	2.0-20.8	***
**Usual Source of Care**			<0.001			<0.001			<0.001			<0.001
Yes	82.7	81.0-84.3		93.5	92.6-94.3		51.7	49.5-53.9		10.0	8.9-11.4	
No	57.1	47.5-66.2		68.8	63.8-73.5		20.6	15.5-26.8		2.0	0.75-5.2	***

FOBT, fecal occult blood test; CI, confidence interval;

Mammography in past 2 years, women age > 39 home FOBT, adult workers in past year, age > 49 screening endoscopy, adult workers within past 5 years for flexible sigmoidoscopy or 10 years for colonoscopy.

*PM, predicted marginals from multivariate logistic models including all variables in Table 3.

p** values are based on an overall Wald F Chi Square test for association from multivariate logistic regression models.

*** relative standard error is greater than 30%, interpret estimate with caution.

Among working women, we saw a small but statistically significant difference in Pap test reporting by paid sick leave status (91.9% vs. 89%, *P* < 0.04). A significant difference in reported Pap testing was also seen for age with the highest proportion of Pap tests being reported by the youngest workers (aged 18–29 years) when compared with all other age groups (p < 0.001). Widowed or divorced workers were less likely to report having had a Pap test (88.2%) than married or partnered workers (92.0%), (*P* < 0.01). In addition, workers with a usual source of care (93.5%; 95% CI, 92.6–94.3) were more likely to have reported a Pap test compared with workers without a usual source of care (68.8%; 95% CI, 63.8–73.5). No associations with Pap testing were seen for education, poverty ratio, health insurance, or race/ethnicity.

A similar pattern was observed for endoscopy reporting. A larger proportion of workers with paid sick leave reported having had an endoscopy (52.5%; 95% CI, 49.9–55.0) than workers who lacked sick leave (43.5%; 95% CI 40.1–47.1). Higher proportions of workers aged 60–69 years or older reported endoscopy compared with those aged 50–59 years (*P* < 0.001). Married workers were significantly more likely to have had an endoscopy (51.7%; 95% CI, 49.0–54.3) than workers who were widowed or divorced (44.8%; 95% CI, 41.4–48.2). Having a usual source of care was significantly associated with reporting an endoscopy (*P* < 0.001). No association was found between education, poverty ratio, health insurance or race/ethnicity and endoscopy. Only age and marital status were statistically and significantly associated with home FOBT. Workers aged 50–59 years were less likely to have reported an FOBT home test than workers aged 60–69 years (*P* < 0.001) or workers 70 years of age and older (*P* = 0.02). Contrary to results for other cancer testing, widowed or divorced workers were more likely to report a home FOBT test (*P* = 0.01). Finally, workers with a usual source of care were more likely than to report a home FOBT than workers without a usual source of care (10.0%; 95% CI, 8.9–11.4 vs. 2.0%; 95% CI, 0.75–5.2).

Table 4 presents results for medical-care-seeking among working men and women. The overall proportion of workers reporting having seen a doctor during the prior year in any setting was higher than the proportion of workers who had at least one physician visit in an office or clinic setting. Only sick leave, age, and marital status significantly predicted having seen a doctor during the prior year or having had at least one physician visit. Workers with sick leave were more likely to have had at least one physician visit in an office or clinic (68.4%; 95% CI, 66.9–69.8) than those without sick leave (59.2%; 95% CI 57.3–61.0). A similar relationship was observed for workers seeking medical care in any setting including an emergency room. As expected, older workers (60-69 years of age and 70 years of age or older) were more likely to report having seen a physician at least once in a clinic or office than workers 50-59 years of age (83.4%; 95% CI, 80.4–86.1 and 83.2%; 95%CI 76.4–88.4 vs. 72.1%; 95% CI, 69.8–74.4). This relationship was also observed for having seen a doctor during the prior year in any setting. Widowed or divorced workers reported the lowest proportions of medical care-seeking as compared with married or partnered workers. No significant relationship was seen for education, poverty level, race/ethnicity or poverty level and medical care seeking.

**Table 4:** Adjusted population percentages and 95% CIs of U.S. workers visiting a physician, NHIS 2008

**Characteristics**	**# Physician Visits (n=11,504)**			**Seen doctor in past year (n=11,533)**		
	**(in clinic or office)**			**(in any setting including ER)**		
	PM*	**95% CI**	P**	PM*	**95% CI**	P**
**Sick leave**						
Yes	68.4	66.9-69.8	<0.001	84.6	82.4-84.9	<0.001
No	59.2	57.3-61.0		72.8	71.2-74.4	
**Age years**						
18-29	55.9	53.1-58.7	<0.001	75.0	72.5-77.4	<0.001
30-39	59.9	57.7-62.0		74.8	72.9-76.6	
40-49	66.7	64.5-69.0		80.1	78.1-82.0	
50-59	72.1	69.8-74.4		84.2	82.2-86.1	
60-69	83.5	80.4-86.1		93.4	91.5-94.9	
70+	83.2	76.4-88.4		93.5	89.2-96.2	
**Race/ethnicity**						
Hispanic	63.8	61.4-66.1	0.54	79.2	77.1-81.2	0.99
Non-Hispanic White	65.1	63.5-66.7		79.5	78.2-80.2	
Non-Hispanic Black	64.2	61.3-67.0		79.4	76.8-81.8	
Non-Hispanic Asian	67.9	63.4-72.0		79.9	76.1-83.2	
Non-Hispanic Other	63.4	51.7-73.6		77.9	66.9-86.1	
**Marital status**						
Never married	64.0	61.8-66.2	0.01	78.1	76.0-80.0	<0.001
Married/partnered	65.8	64.2-67.4		80.1	79.3-82.0	
Widowed/Divorced	61.4	58.5-64.2		75.9	73.4-78.2	
**Education**			0.48			0.21
< high school	66.2	64.2-68.2		80.9	79.2-82.5	
High school/GED	64.3	62.0-66.6		79.2	77.1-81.2	
Some college	64.4	61.9-66.8		79.1	76.8-81.3	
College graduate	63.5	60.9-66.0		78.5	76.2-80.6	
missing	64.7	60.9-68.4		77.7	74.2-80.8	
**Poverty ratio**						
< 100%	64.1	61.1-67.1	0.91	80.6	77.6-83.4	0.11
100% to <200%	65.6	62.8-68.4		80.0	77.6-82.2	
200% to <400%	64.2	62.0-66.3		80.8	78.9-82.6	
400% or more	65.1	63.0-67.0		77.7	76.0-79.4	
Unknown	65.1	62.5-67.6		79.2	77.0-81.3	
**Health insurance**						
Private	66.2	64.3-68.0	0.26	80.6	78.6-82.4	0.12
Public only	63.6	61.3-65.8		79.2	77.1-81.2	
Public and Private	64.2	62.1-66.3		79.1	76.3-79.9	
Not covered	65.5	62.6-68.2		79.5	77.1-81.8	
Unknown	58.6	49.2-67.2		86.1	78.8-91.1	

*PM, predicted marginal from logistic regression model using all variables in Table 4.

p** values are based on an overall Wald Chi Square test for association from multivariate logistic regression models.

ER is Emergency Room.

## Conclusions

Out of an estimated U.S. working population of 127 million in 2008, more than 48 million (38%) lack paid sick leave. Approximately 60% of private-sector workers and more than 80% of state and local government workers had paid sick leave. Our analysis shows that it was workers in service or production occupations, those in the private sector, and those in smaller firms with fewer years on the job who were less likely to report having sick leave. Furthermore, our results from this nationally representative sample demonstrate that sick leave could be a significant barrier to cancer testing and medical care seeking.

Both unadjusted and adjusted proportion of workers undergoing mammography, Pap test, endoscopy and medical care-seeking were significantly higher for those with paid sick leave than those who lacked paid sick leave. It was only for home FOBT that we did not see an association with paid sick leave. Compared with endoscopy which requires contact with a physician and time away from work, testing for blood in the stool with an FOBT test kit is performed at home. In addition, the proportion reporting home FOBT was much smaller than the proportion reporting endoscopy.

Screening behavior is affected by a myriad of factors that vary within different populations. We adjusted for sociodemographic factors that have been shown to be barriers or facilitators of cancer testing or medical care-seeking in the United States. Race/ethnicity, education, age, household income, marital status, usual source of care and health-care coverage have been associated with colorectal cancer screening [10,12,13], mammography [9,13], and Pap testing [13] in population-based surveys, including the NHIS and a random sample of Medicare beneficiaries [22]. Our study population, which included only U.S. working men and women, is likely to differ in important ways from the U.S. population as a whole or the Medicare population. Consistent with previous research, we also reported a significant contribution of age and marital status to models of cancer screening or medical care-seeking as outcomes, but saw no significant differences in cancer screening by health insurance status or poverty. This could be due to a population of working men and women having less variability in insurance status and poverty level than a general population. Among working adults, lack of paid sick leave may pose a more significant barrier to cancer testing and medical care-seeking than lack of insurance or poverty.

This analysis has some limitations. For example, data are based on self-report and respondents may have incorrectly reported their screening use and the timing of that screening. A recent meta-analysis of the accuracy of self-reports of cancer screening concluded that national survey data overestimate the prevalence of screening and mask disparities by race and ethnicity because of differences in reporting accuracy [23]. In addition, the survey seeks information only on paid sick leave and no other leave such as paid personal or annual leave, and the survey does not capture any restrictions on the use of sick leave for preventive health care. Workers may have personal leave or vacation leave but may not consider or report these categories as paid sick leave. Thus, we may have underestimated the proportion of workers with leave that could be counted for cancer screening. However, our estimates of worker’s access to paid sick leave were similar to the Bureau of Labor Statistics (BLS) estimates of 61% for private-industry workers and 89% of state and local workers during 2008 [24]. Differences are primarily due to differences in survey design. Whereas the NHIS is a survey of randomly chosen individuals from households who are representative of the noninstitutionalized U.S. population, the BLS estimates are obtained from the National Compensation Survey, an employer-based survey representing a random selection of establishments chosen from state unemployment insurance records [25].

Barriers to cancer screening and routine medical care-seeking involve a complex web of individual, community, health care system and societal characteristics. In the working population, a person’s occupation is the source of his or her income and medical insurance coverage, and of other benefits such as paid sick leave, worker’s compensation, paid vacation, and retirement benefits [26]. In short, a person’s occupation is the source of some of the most critical elements determining their health and well-being. And in the United States, access to these benefits is largely determined by the type of occupation. The percentage of workers with access to paid sick leave is lowest among service workers, workers in construction and maintenance, transportation workers, and part-time workers, and highest among managers and professional workers. This occupational structure disproportionately affects women who are more likely to be low-wage and part-time workers [27].

Lack of paid sick leave can be considered within the category of out-of-pocket costs for medical care. Those without sick leave who take work time off for preventive services may lose pay. High deductibles and other forms of cost sharing have been associated with underuse of preventive services [28,29], specifically colorectal cancer screening [30] and mammography [31,32]. Lack of paid sick leave appears to be a potential barrier to obtaining needed medical care and a societal benefit that is potentially amenable to change.

## Abbreviations

NHIS, National Health Interview Survey; CI, Confidence interval; FOBT, Fecal occult blood test; NCHS, National Center for Health Statistics; FMLA, Family and Medical Leave Act; USPSTF, United States Preventive Services Task Force.

## Competing interests

The authors declare that they have no competing interests.

## Authors’ contributions

LAP, ZB, AS and MCW designed the study. AS and ZB conducted the statistical analyses. LAP, AS, ZB and MCW contributed to the interpretation of the data. LAP drafted the manuscript with contributions from AS, ZB and MCW. LAP, AS, ZB and MCW read and approved the final manuscript.

The findings and conclusions in this report are those of the authors and do not necessarily represent the official position of the Centers for Disease Control and Prevention.

## Pre-publication history

The pre-publication history for this paper can be accessed here:

http://www.biomedcentral.com/1471-2458/12/520/prepub
